# Supplementary figures and images for: Differential requirements for mitochondrial electron transport chain components in the adult murine liver
Source: eLife. 2022 Sep 26;11:e80919. doi: 10.7554/eLife.80919 (PMC9648974; doi:10.7554/eLife.80919)

Fig. 1B - Ndufa9

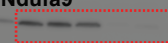

Fig. 1B -  $\beta$ 2M

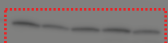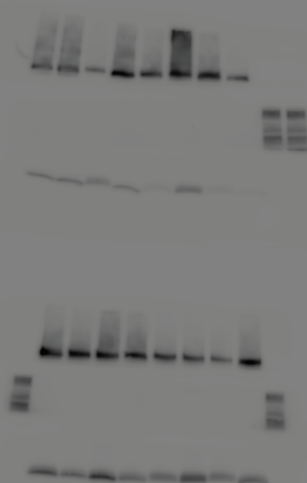

Supplement: Figure 1—source data 1. — Full gel images and original image files for western blots in Figure 1. [file elife-80919-fig1-data1.zip › Figure 1 - source data 1/Figure 1 - source data file 1.pdf]

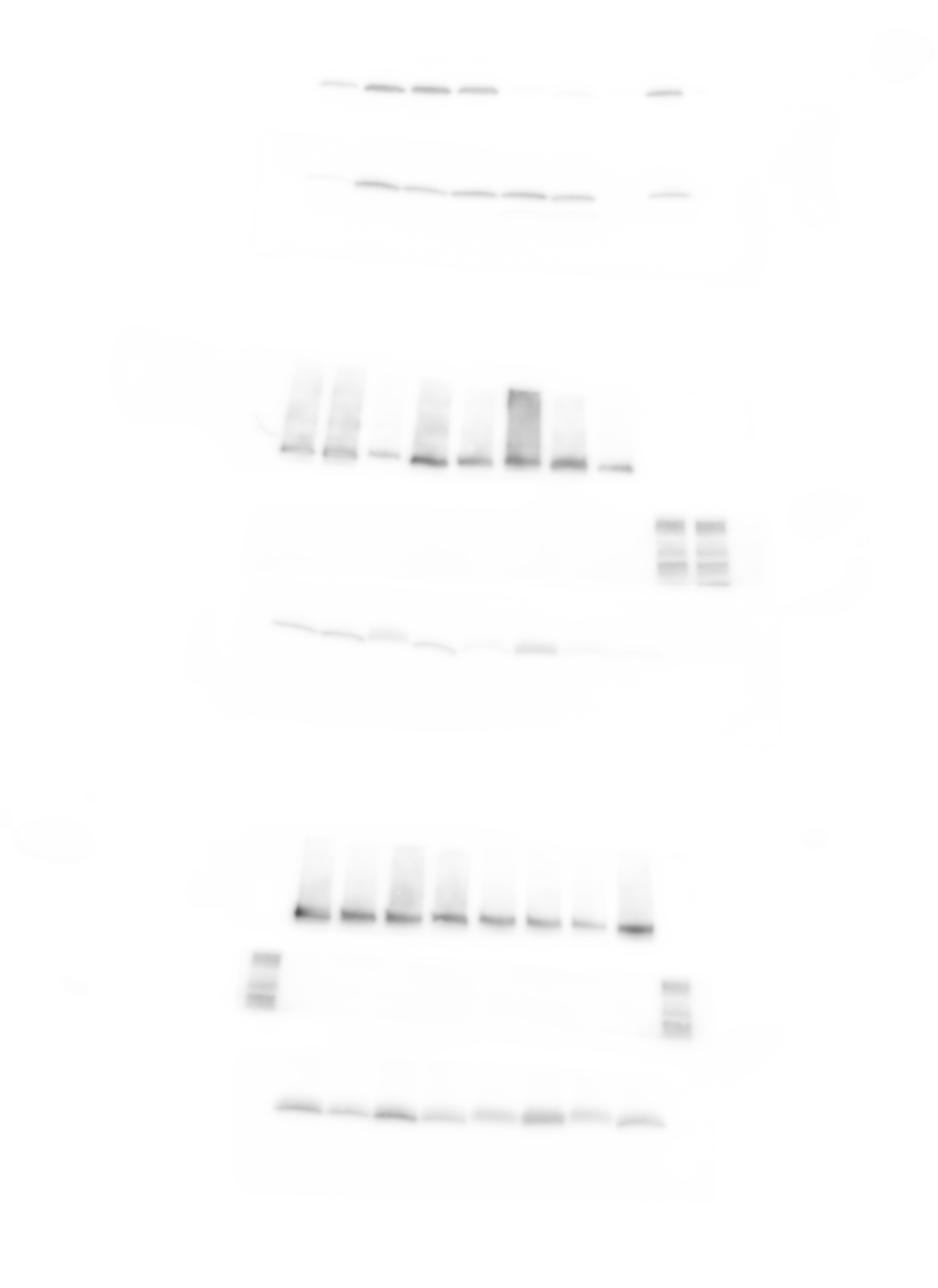

Supplement: Figure 1—source data 1. — Full gel images and original image files for western blots in Figure 1. [file elife-80919-fig1-data1.zip › Figure 1 - source data 1/Figure 1B_Ndufa9_B2M.tif]

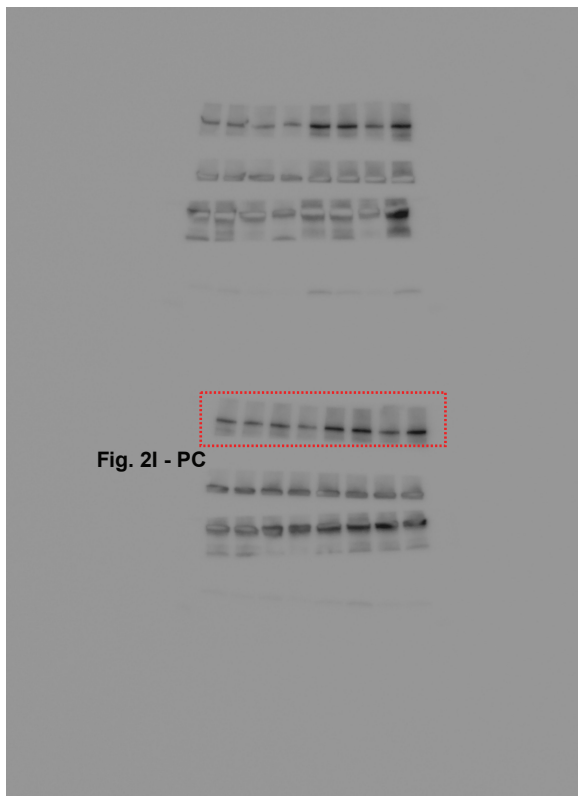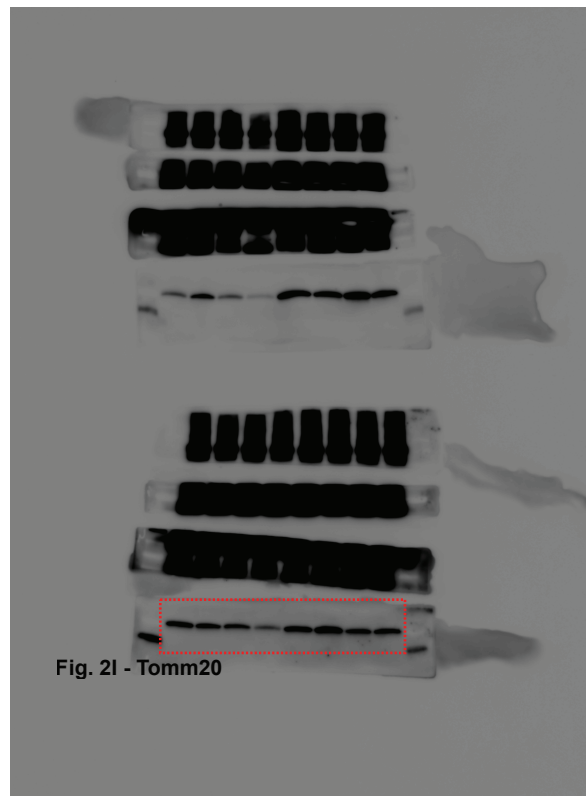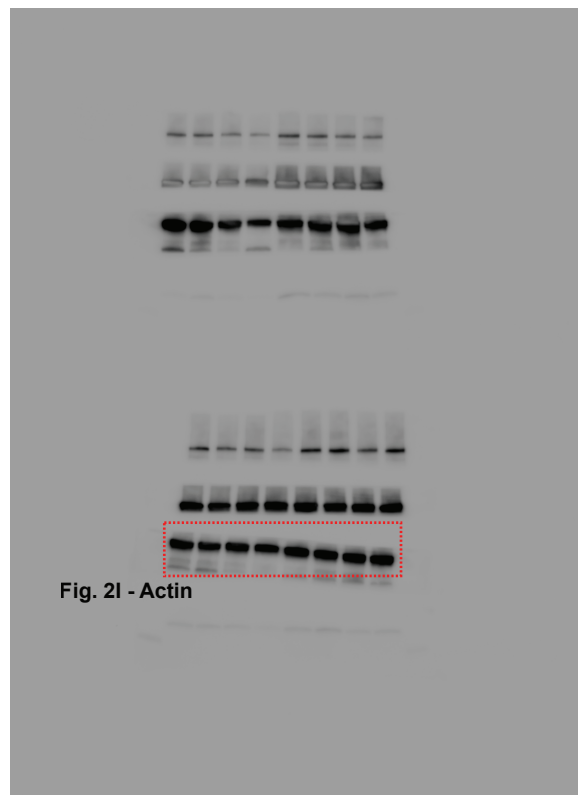

Supplement: Figure 2—source data 1. — Full gel images and original image files for western blots in Figure 2. [file elife-80919-fig2-data1.zip › Figure 2 - source data 1/Figure 2 - source data file 1.pdf]

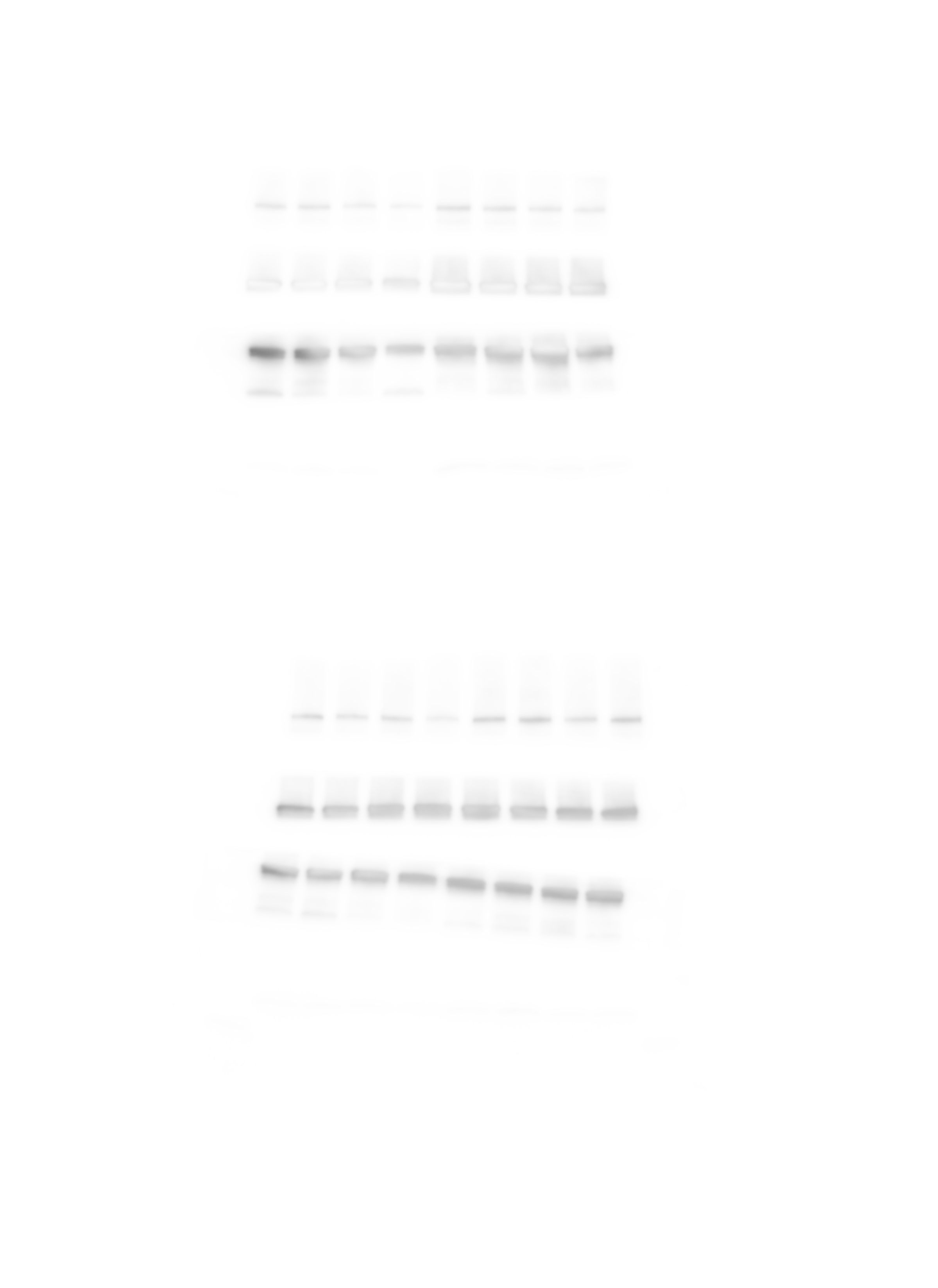

Supplement: Figure 2—source data 1. — Full gel images and original image files for western blots in Figure 2. [file elife-80919-fig2-data1.zip › Figure 2 - source data 1/Figure2I_Actin.tif]

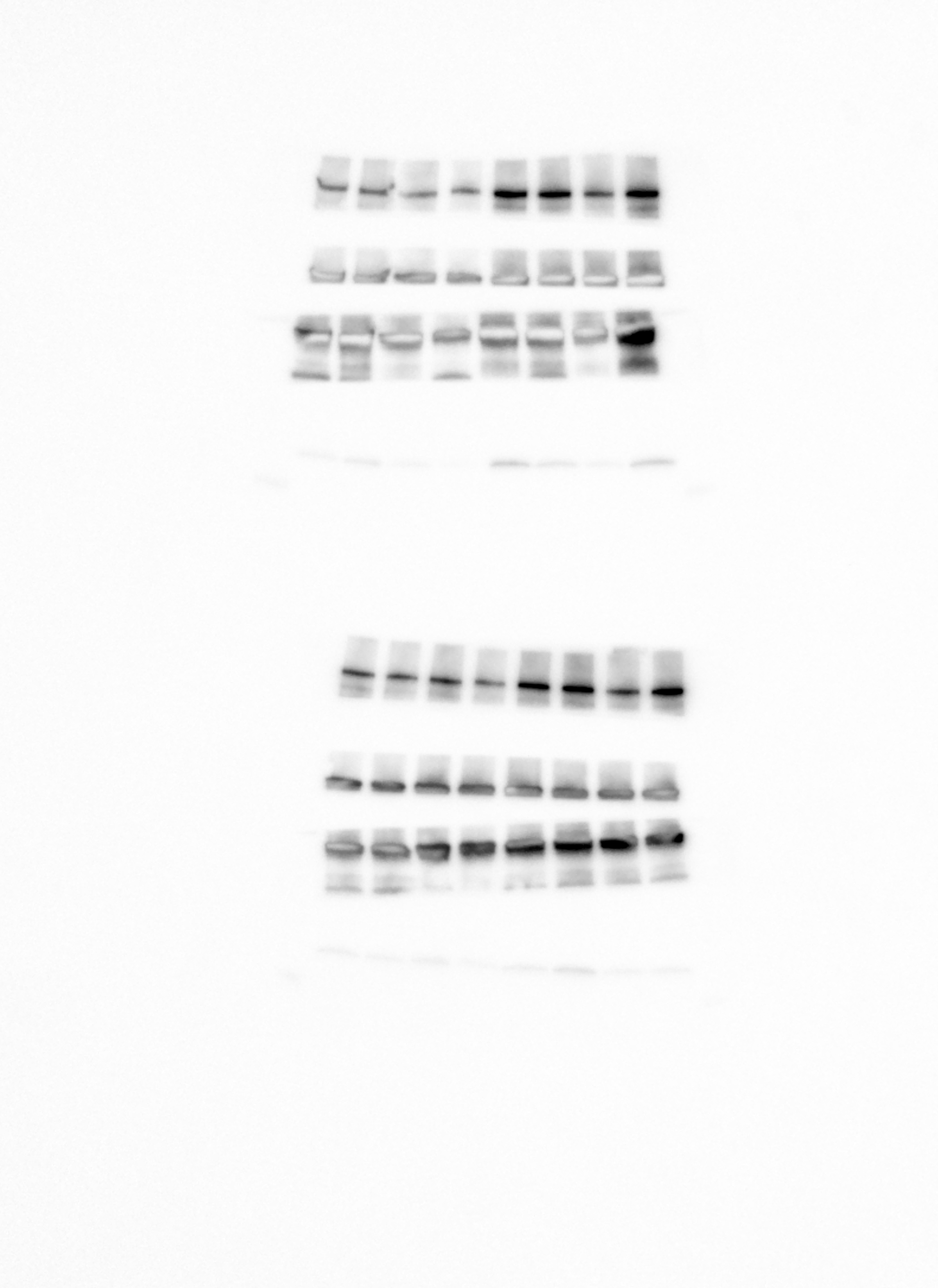

Supplement: Figure 2—source data 1. — Full gel images and original image files for western blots in Figure 2. [file elife-80919-fig2-data1.zip › Figure 2 - source data 1/Figure2I_PC.tif]

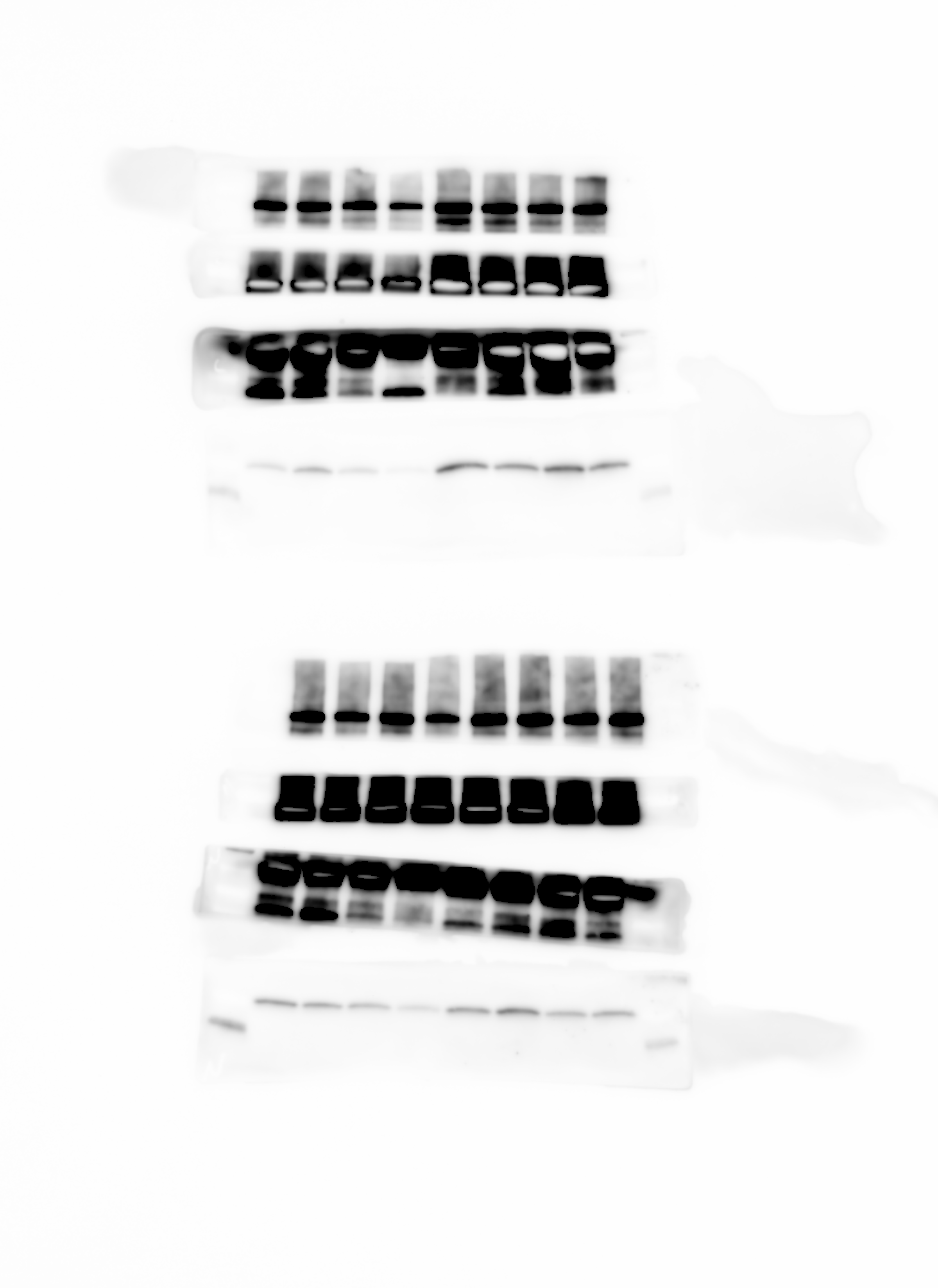

Supplement: Figure 2—source data 1. — Full gel images and original image files for western blots in Figure 2. [file elife-80919-fig2-data1.zip › Figure 2 - source data 1/Figure2I_Tomm20.tif]

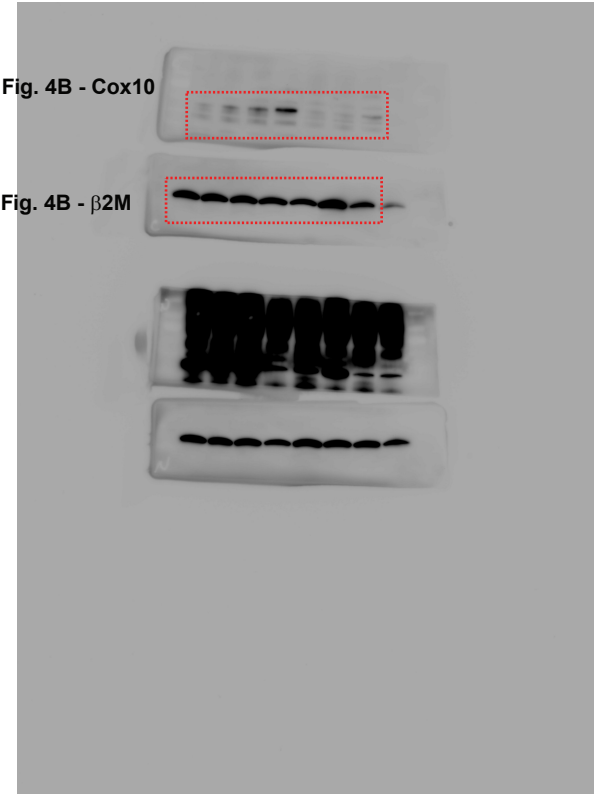

Supplement: Figure 4—source data 1. — Full gel images and original image files for western blots in Figure 4. [file elife-80919-fig4-data1.zip › Figure 4 - source data 1/Figure 4 - source data file 1.pdf]

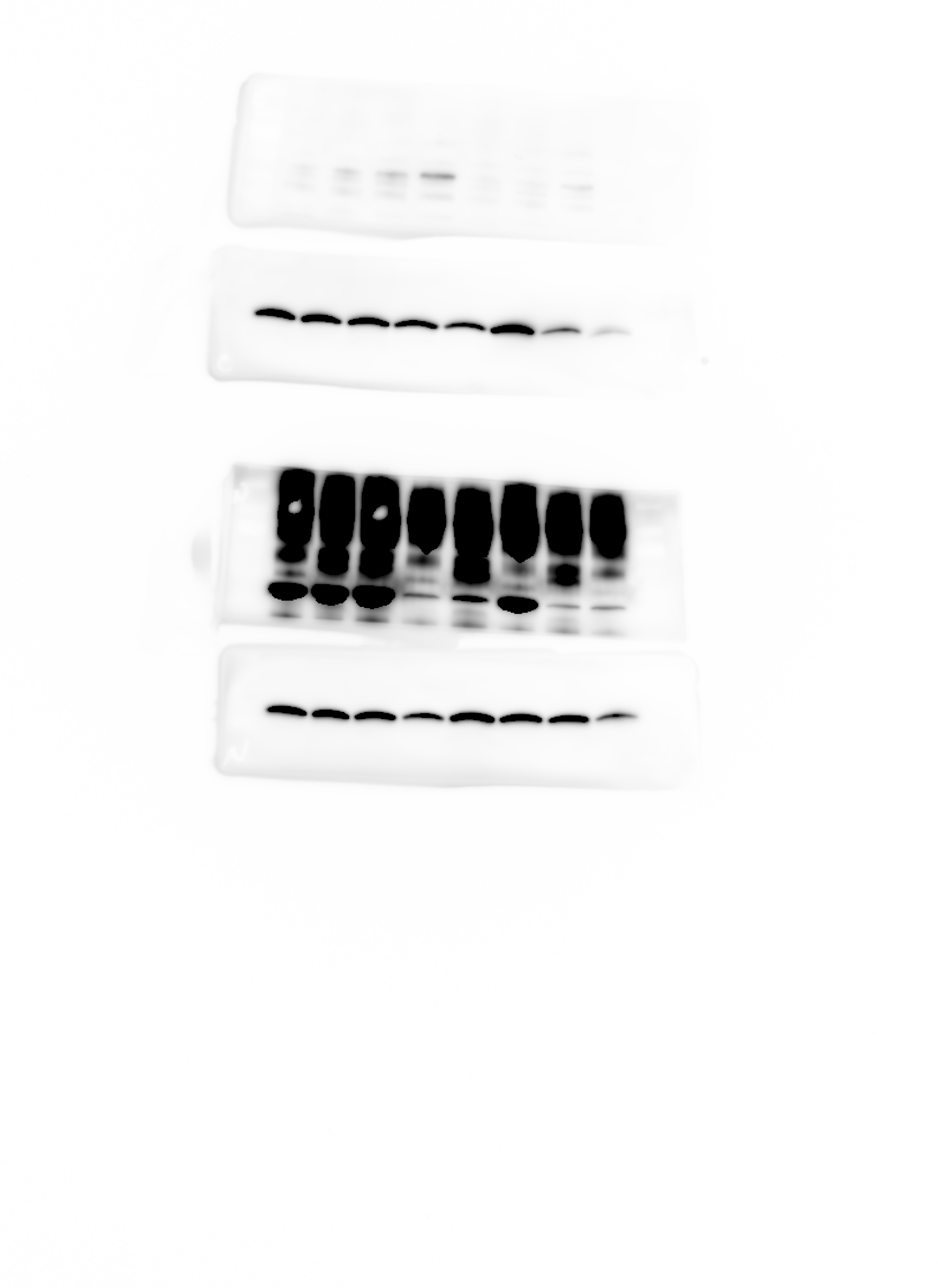

Supplement: Figure 4—source data 1. — Full gel images and original image files for western blots in Figure 4. [file elife-80919-fig4-data1.zip › Figure 4 - source data 1/Figure 4B_Cox10_B2M.tif]

Fig. 5I - PC

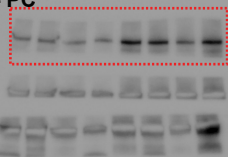

Fig. 5I - Tomm20

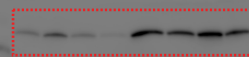

Fig. 5I - Actin

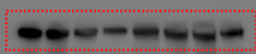

Supplement: Figure 5—source data 1. — Full gel images and original image files for western blots in Figure 5. [file elife-80919-fig5-data1.zip › Figure 5 - source data 1/Figure 5 - source data file 1.pdf]
